# Redox biology and therapeutics in chronic lung disease

**DOI:** 10.1016/j.redox.2020.101579

**Published:** 2020-05-11

**Authors:** Victor J. Thannickal

**Affiliations:** Division of Pulmonary, Allergy, and Critical Care Medicine, Department of Medicine, University of Alabama at Birmingham, 1900 University Blvd, THT 541E, Birmingham, AL, 35294, USA

Chronic lung diseases, lung cancer and lower respiratory infections contribute to over 8 million deaths each year, making respiratory-related disorders the second leading cause of mortality worldwide [1,2]. The primary function of the lung is gas-exchange, which allows for oxygen (O_2_)-rich air to diffuse across alveolar-capillary units of the lower airways; simultaneously, the metabolic waste product, carbon dioxide (CO_2_), diffuses out of the circulation and is expelled via the airways to the atmosphere [3]. Thus, the mammalian respiratory system essentially supplies O_2_ to serve as the final electron acceptor in the mitochondrial electron chain, coupled to oxidative phosphorylation. In addition to aerobic metabolism, however, O_2_ and its reduced intermediates referred to as reactive oxygen species (ROS) participate as essential mediators in biosynthetic pathways, host defense, cellular signaling and oxidative stress [4]. It is not surprising, then, that ROS are not only recognized as essential metabolites in normal physiological functions such as maintenance of tissue homeostasis and adaptive stress responses, but are also implicated in myriad pathological processes that lead to chronic diseases affecting diverse organs, including the lung.

Chronic lung diseases have traditionally been divided into two major classes – obstructive and restrictive. This classification is based primarily on pulmonary function testing that assesses, among other parameters, an individual's ability to expel inspired air, and measurement of forced expiratory volume in 1 s (FEV1); an obstructive pattern is characterized by a reduced FEV1 relative to total forced expiratory volume (FVC). Such a pattern is most commonly encountered in pathological conditions when there is narrowing of the conducting airways, enlargement of alveolar sacs and loss of elastic recoil, diminished expiratory muscle strength/leverage or a combination of these. Chronic obstructive pulmonary disease (COPD) and asthma account for the vast majority of the obstructive lung disorders in clinical medicine. In contrast, restrictive lung disorders are characterized by reduced capacity to move inspired air into the lung and a reduction in lung volumes; in such cases, both FEV1 and FVC are reduced proportionally, without much change in the FEV1/FVC ratio. Although diseases affecting the musculoskeletal system and the pleural lining/covering of the lung can manifest in restrictive physiology, the majority of restrictive lung disorders encountered in clinical practice are interstitial lung diseases, the most common of which is idiopathic pulmonary fibrosis (IPF). It should be recognized, however, that physiological patterns of obstruction and restriction can co-exist in individual subjects; moreover, the anatomic site of pathological involvement (e.g. upper vs. lower airway) or the nature of the disease process cannot be inferred simply based on these physiological designations.

There is growing recognition that similar biological processes or pathological endotypes may contribute to disease development and progression in diverse obstructive and restrictive lung disorders, in particular COPD and IPF. These shared pathological endotypes include cellular senescence, apoptosis, fibrosis, autoimmunity, and aberrant cell differentiation/fate. In this special issue, leading experts in redox biology and chronic lung diseases discuss the emergence of these pathological endotypes in COPD and IPF, and the mechanisms of how redox signaling and oxidative stress contribute to the initiation and/or progression of these lung disorders. Importantly, each of the articles in this special issue address therapeutic opportunities to modulate redox signaling and mitigate oxidative stress in chronic lung disease.

Peter Barnes provides an overview of the role of oxidative stress in many of the pathological hallmarks of COPD, namely unremitting inflammation, accelerated aging, DNA damage responses, autoimmunity and corticosteroid resistance [5]. After providing a historical context for the past failure of some antioxidant strategies, the author posits that effective drugs such as superoxide dismutase mimetics, NADPH oxidase (Nox) inhibitors, mitochondria-targeted antioxidants and Nrf2 activators hold promise for the future.

Irfan Rahman and colleagues remind us that, by virtue of its function in gas-exchange, the lungs are inevitably exposed to airborne pathogens, particulates, cigarette smoke and other toxicants [6]. These authors provide an incisive description of mitochondria-endoplasmic reticulum (ER) crosstalk as a generalized stress response to environmental challenges. Interestingly, this mitochondria-ER crosstalk regulates the circadian clock and modulates the amplitude of circadian protein oscillations. The role of mitochondrial dysfunction and cellular senescence in accelerated aging and COPD pathogenesis is also discussed.

Yvonne Janssen-Heininger and colleagues expand on the theme of ER stress by providing evidence that dysregulation of S-glutathionylation is associated with ER stress in settings of chronic lung diseases [7]. They provide a discerning review of glutathione biochemistry, processes that regulate S-glutathionylation, and how this contributes the pathogenesis of asthma, COPD and IPF. The authors also provide rationale for developing small molecules or biologics that harness S-glutathionylation chemistry for the treatment of chronic lung disease.

In two different and complementary articles, Ann Mora [8] and Brent Carter [9] and their coauthors provide a detailed and insightful review of metabolic reprogramming and mitochondrial dysfunction in three cell types, alveolar epithelial cells, fibroblasts, and macrophages, linked to disease pathogenesis in IPF. Both articles highlight the complexity and divergent regulation of mitochondrial biogenesis and turnover in different cell types that govern specific pro-fibrotic phenotypes. While Mora and colleagues focus on critical metabolic hubs that may be targeted for the development of novel anti-fibrotic drugs, Carter and colleagues postulate that modulation of mitochondrial quality control may be worthy of future exploration.

In addition to mitochondria, the Nox family of enzymes are bona fide ROS-generators; in fact, the production of ROS appears it be the primary function of this unique family of proteins [[10], [11]]. In their article, Kosuke Kato and Louise Hecker discuss the role of Nox enzymes in age-related susceptibility to pulmonary fibrosis, with a particular focus on Nox4 [12]. These authors review the evidence for Nox4 in the induction of myofibroblast senescence, apoptosis resistance and loss of cellular plasticity; they offer caveats and opportunities for Nox-targeted drug discovery and development.

The respiratory system is uniquely challenged by exogenous (airborne) sources of oxidants in addition to endogenous capacity to generate ROS for purposes of cell signaling and regulation. Normal physiological functions of ROS may be subverted to pathological roles in the context of chronic lung disease and aging, when endogenous antioxidant capacity wanes [13]. Thus, the distinction between redox signaling and oxidative stress may become blurred as we age [[14], [15]]. These unique challenges also offer exciting opportunities for the development of redox-modulatory agents, Nox inhibitors, and mitochondrial-targeted drugs for chronic lung diseases.

## References

[1] Li X., Cao X., Guo M., Xie M., Liu X. (2020). Trends and risk factors of mortality and disability adjusted life years for chronic respiratory diseases from 1990 to 2017: systematic analysis for the Global Burden of Disease Study 2017. BMJ.

[2] Global Health Estimates (2018). 2016: Deaths by Cause, Age, Sex, by Country and by Region, 2000-2016 Geneva.

[3] Thannickal V.J. (2009). Oxygen in the evolution of complex life and the price we pay. Am. J. Respir. Cell Mol. Biol..

[4] Thannickal V.J., Fanburg B.L. (2000). Reactive oxygen species in cell signaling. Am. J. Physiol. Lung Cell Mol. Physiol..

[5] Barnes P.J. (2020). Oxidative stress-based therapeutics in COPD. Redox Biol..

[6] Manevski M., Muthumalage T., Devadoss D., Sundar I.K., Wang Q., Singh K.P., Unwalla H.J., Chand H.S., Rahman I. (2020). Cellular stress responses and dysfunctional Mitochondrial-cellular senescence, and therapeutics in chronic respiratory diseases. Redox Biol..

[7] Janssen-Heininger Y., Reynaert N.L., van der Vliet A., Anathy V. (2020). Endoplasmic reticulum stress and glutathione therapeutics in chronic lung diseases. Redox Biol..

[8] Bueno M., Calyeca J., Rojas M., Mora A.L. (2020). Mitochondria dysfunction and metabolic reprogramming as drivers of idiopathic pulmonary fibrosis. Redox Biol..

[9] Larson-Casey J.L., He C., Carter A.B. (2020). Mitochondrial quality control in pulmonary fibrosis. Redox Biol..

[10] Bernard K., Hecker L., Luckhardt T.R., Cheng G., Thannickal V.J. (2014). NADPH oxidases in lung health and disease. Antioxidants Redox Signal..

[11] Hecker L., Vittal R., Jones T., Jagirdar R., Luckhardt T.R., Horowitz J.C., Pennathur S., Martinez F.J., Thannickal V.J. (2009). NADPH oxidase-4 mediates myofibroblast activation and fibrogenic responses to lung injury. Nat. Med..

[12] Kato K., Hecker L. (2020). NADPH oxidases: Pathophysiology and therapeutic potential in age-associated pulmonary fibrosis. Redox Biol..

[13] Hecker L., Logsdon N.J., Kurundkar D., Kurundkar A., Bernard K., Hock T., Meldrum E., Sanders Y.Y., Thannickal V.J. (2014). Reversal of persistent fibrosis in aging by targeting Nox4-Nrf2 redox imbalance. Sci. Transl. Med..

[14] Thannickal V.J., Zhou Y., Gaggar A., Duncan S.R. (2014). Fibrosis: ultimate and proximate causes. J. Clin. Invest..

[15] Thannickal V.J. (2010). Aging, antagonistic pleiotropy and fibrotic disease. Int. J. Biochem. Cell Biol..

